# Supplementary material for: Genomic Landscape of Early-Onset and Familial Latin American Parkinson’s Patients
Source: medRxiv. 2026 Sep 22:2026.09.16.26359514. Preprint. [Version 1] doi: 10.64898/2026.09.16.26359514 (PMC13622300; doi:10.64898/2026.09.16.26359514)
Supplement: Supplementary file 1 [file media-2.docx]

**Supplementary methods**

**Variant annotation**

Identified SNVs and indels in the genes of interest were annotated using the Ensembl Variant Effect Predictor (VEP) tool [(1)](https://paperpile.com/c/UiR2NO8B/EPhA). Additional plug-ins to provide Combined Annotation Dependent Depletion (CADD) [(2)](https://paperpile.com/c/UiR2NO8B/gCxX) scores and Genome Aggregation Database (gnomAD, version 4.1) [(3)](https://paperpile.com/c/UiR2NO8B/FPaT) allele frequencies were utilized.

Moreover, annotations with the snpEff tool to retrieve functional consequences of the variants were performed. The first instances of functional characterization per variant were selected, including predicted impact severity and functional consequences. Additionally, we used the ANNOVAR [(4)](https://paperpile.com/c/UiR2NO8B/PL1r) tool to assign rsIDs and retrieve variant pathogenicity classifications from ClinVar (version 20250721) [(5)](https://paperpile.com/c/UiR2NO8B/kRg0); in silico prediction scores from dbNSFP (version 47 academic) [(6)](https://paperpile.com/c/UiR2NO8B/aNDI) were also used. To characterize the deleteriousness of the variants, CADD score, MutationTaster [(7)](https://paperpile.com/c/UiR2NO8B/tHyn), PrimateAI [(8)](https://paperpile.com/c/UiR2NO8B/qFEi), AlphaMissense [(9)](https://paperpile.com/c/UiR2NO8B/MCiW), SIFT [(10)](https://paperpile.com/c/UiR2NO8B/bZpB), and PolyPhen2 [(11)](https://paperpile.com/c/UiR2NO8B/kQWO) predictions were selected.

Lastly, to characterize the gene-disease relationships associated with the identified variants, the Online Mendelian Inheritance in Man database (OMIM) [(12)](https://paperpile.com/c/UiR2NO8B/c8JC) was utilized to retrieve the Mendelian phenotypes and corresponding MIM numbers associated with each gene harboring a variant.

**References**

[1.	McLaren W, Gil L, Hunt SE, et al. The Ensembl Variant Effect Predictor. Genome Biol 2016;17(1):122. https://doi.org/10.1186/s13059-016-0974-4](https://paperpile.com/b/UiR2NO8B/EPhA)

[2.	Rentzsch P, Witten D, Cooper GM, Shendure J, Kircher M. CADD: predicting the deleteriousness of variants throughout the human genome. Nucleic Acids Res 2019;47(D1):D886–D894. https://doi.org/10.1093/nar/gky1016](https://paperpile.com/b/UiR2NO8B/gCxX)

[3.	Karczewski KJ, Francioli LC, Tiao G, et al. The mutational constraint spectrum quantified from variation in 141,456 humans. Nature 2020;581(7809):434–443. https://doi.org/10.1038/s41586-020-2308-7](https://paperpile.com/b/UiR2NO8B/FPaT)

[4.	ANNOVAR: Functional annotation of genetic variants from next-generation sequencing data Nucleic Acids Research. Nucleic Acids Research.](https://paperpile.com/b/UiR2NO8B/PL1r)

[5.	Landrum MJ, Lee JM, Riley GR, et al. ClinVar: public archive of relationships among sequence variation and human phenotype. Nucleic Acids Res 2014;42(Database issue):D980-5. https://doi.org/10.1093/nar/gkt1113](https://paperpile.com/b/UiR2NO8B/kRg0)

[6.	Liu X, Jian X, Boerwinkle E. dbNSFP: a lightweight database of human nonsynonymous SNPs and their functional predictions. Hum Mutat 2011;32(8):894–899. https://doi.org/10.1002/humu.21517](https://paperpile.com/b/UiR2NO8B/aNDI)

[7.	Steinhaus R, Proft S, Schuelke M, et al. MutationTaster2021. Nucleic Acids Res 2021;49(W1):W446–W451. https://doi.org/10.1093/nar/gkab266](https://paperpile.com/b/UiR2NO8B/tHyn)

[8.	Parry D, Bosc T, Hamp T, et al. PrimateAI-3D outperforms AlphaMissense in real-world cohorts. *medRxiv*. https://doi.org/10.1101/2024.01.12.24301193](https://paperpile.com/b/UiR2NO8B/qFEi)

[9.	Cheng J, Novati G, Pan J, et al. Accurate proteome-wide missense variant effect prediction with AlphaMissense. Science 2023;381(6664):eadg7492. https://doi.org/10.1126/science.adg7492](https://paperpile.com/b/UiR2NO8B/MCiW)

[10.	Ng PC, Henikoff S. SIFT: Predicting amino acid changes that affect protein function. Nucleic Acids Res 2003;31(13):3812–3814. https://doi.org/10.1093/nar/gkg509](https://paperpile.com/b/UiR2NO8B/bZpB)

[11.	Adzhubei IA, Schmidt S, Peshkin L, et al. A method and server for predicting damaging missense mutations. Nat Methods 2010;7(4):248–249. https://doi.org/10.1038/nmeth0410-248](https://paperpile.com/b/UiR2NO8B/kQWO)

[12.	Hamosh A, Scott AF, Amberger JS, Bocchini CA, McKusick VA. Online Mendelian Inheritance in Man (OMIM), a knowledgebase of human genes and genetic disorders. Nucleic Acids Res 2005;33(Database issue):D514-7. https://doi.org/10.1093/nar/gki033](https://paperpile.com/b/UiR2NO8B/c8JC)
